# Supplementary material for: Cytosolic aggregation of mitochondrial proteins disrupts cellular homeostasis by stimulating the aggregation of other proteins
Source: eLife. 2021 Jul 20;10:e65484. doi: 10.7554/eLife.65484 (PMC8457837; doi:10.7554/eLife.65484)
Supplement: Figure 1—source data 1. — Protein sequences and information about mitochondrial targeting presequences were acquired from the Saccharomyces Genome Database and verified using Mitofates (Fukasawa et al., 2015) and MitoProt (Claros and Vincens, 1996) software. Protein solubility was analyzed using CamSol (Sormanni et al., 2015) software. Proteins and sequence residues with scores < –1 were poorly soluble and indicated as potential self-assembly hotspots (red). Scores > 1 characterize highly soluble proteins and sequence residues (blue). TMDs: transmembrane domains; IMS: intermembrane space; IM: inner membrane. [file elife-65484-fig1-data1.docx]

| Human  homologs | Protein name  (ORF name) | Localization | Intrinsic residue solubility along amino acid sequence | Intrinsic solubility score | Mitochondria targeting sequence |
| --- | --- | --- | --- | --- | --- |
|  |  | Amount of TMDs |  |  |  |
| ATP5B | Atp2  (YJR121W) | peripheral IM | 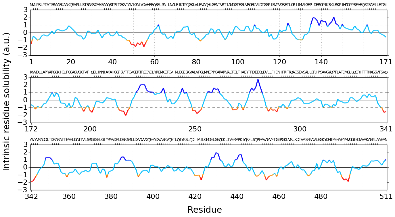 | 0.4783 | N-terminal cleavable presequence |
|  |  | 0 |  |  |  |
| ATP5L | Atp20  (YPR020W) | IM | 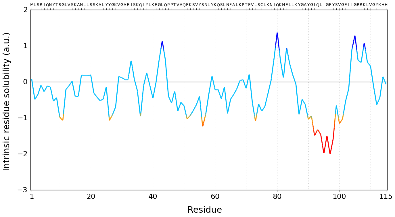 | 0.9556 | N-terminal non-cleavable sequence |
|  |  | 1 |  |  |  |
| COX7C | Cox8  (YLR395C) | IM | 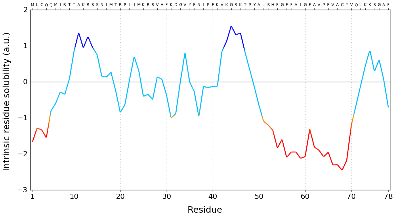 | -0.2763 | N-terminal cleavable presequence |
|  |  | 1 |  |  |  |
| COX6B1 | Cox12  (YLR038C) | IMS | 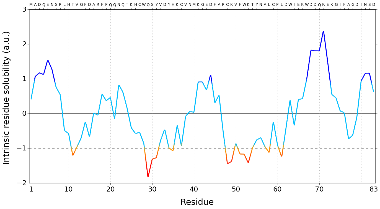 | 1.2939 | Cystein motifs |
|  |  | 0 |  |  |  |
| UQCRFS1 | Rip1  (YEL024W) | IM | 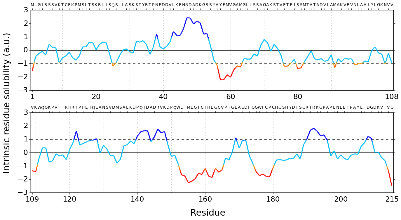 | 0.4783 | N-terminal cleavable presequence |
|  |  | 2 |  |  |  |
| UQCRH | Qcr6  (YFR033C) | IMS | 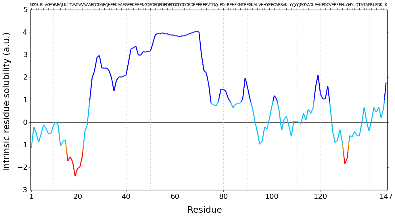 | 4.1091 | Unknown |
|  |  | 0 |  |  |  |
| UQCRQ | Qcr8  (YJL166W) | IM | 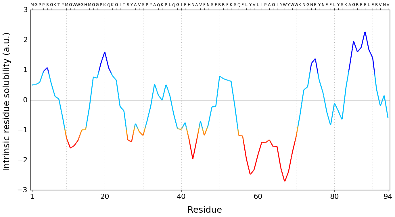 | 0.0079 | N-terminal non-cleavable sequence |
|  |  | 1 |  |  |  |
| UQCRC1 | Cor1  (YBL045C) | IM | 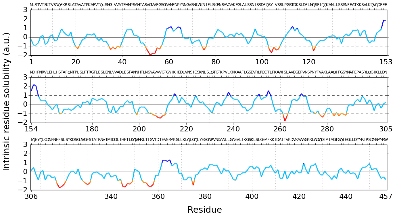 | 0.3800 | N-terminal cleavable presequence |
|  |  | 0 |  |  |  |
