# Supplementary material for: Cytosolic aggregation of mitochondrial proteins disrupts cellular homeostasis by stimulating the aggregation of other proteins
Source: eLife. 2021 Jul 20;10:e65484. doi: 10.7554/eLife.65484 (PMC8457837; doi:10.7554/eLife.65484)
Supplement: Transparent reporting form [file elife-65484-transrepform1.docx]

***eLife’s* transparent reporting form**

We encourage authors to provide detailed information *within their submission* to facilitate the interpretation and replication of experiments. Authors can upload supporting documentation to indicate the use of appropriate reporting guidelines for health-related research (see [EQUATOR Network](http://www.equator-network.org/%20)), life science research (see the [BioSharing Information Resource](https://biosharing.org/)), or the [ARRIVE guidelines](http://www.plosbiology.org/article/info:doi/10.1371/journal.pbio.1000412) for reporting work involving animal research. Where applicable, authors should refer to any relevant reporting standards documents in this form.

If you have any questions, please consult our Journal Policies and/or contact us: [editorial@elifesciences.org](mailto:editorial@elifesciences.org).

**Sample-size estimation**

- You should state whether an appropriate sample size was computed when the study was being designed
- You should state the statistical method of sample size computation and any required assumptions
- If no explicit power analysis was used, you should describe how you decided what sample (replicate) size (number) to use

Please outline where this information can be found within the submission (e.g., sections or figure legends), or explain why this information doesn’t apply to your submission:

No statistical method was used to predetermine sample size. This statement is made in the Materials and methods section, subchapter “Experimental Design”. The standard of minimum 3 biological replicates was used in all experiments, which is also mentioned in the “Experimental Design” section. Experiments with more sample size have information in the legends of individual experiments, for details please see section below.

**Replicates**

- You should report how often each experiment was performed
- You should include a definition of biological versus technical replication
- The data obtained should be provided and sufficient information should be provided to indicate the number of independent biological and/or technical replicates
- If you encountered any outliers, you should describe how these were handled
- Criteria for exclusion/inclusion of data should be clearly stated
- High-throughput sequence data should be uploaded before submission, with a private link for reviewers provided (these are available from both GEO and ArrayExpress)

Please outline where this information can be found within the submission (e.g., sections or figure legends), or explain why this information doesn’t apply to your submission:

The standard of minimum 3 biological replicates has been used (Material and methods, section “Experimental design”. Information on the number of experiments are also provided in the figure legends to Figure 1-5 and Figure 1- Figure supplement 2, Figure 3–figure supplement 1, Figure 3–figure supplement 2, Figure 4–figure supplement 1, Figure 5–figure supplement 1. The number of replicates in RNA-seq data is provided in the Materials and Methods chapter, section RNA-seq – sample preparation.

The RNA-seq data were deposited and are available in GEO repository under the accession number GSE147284, as indicated in the Materials and methods chapter, section RNA-seq – analysis.

**Statistical reporting**

- Statistical analysis methods should be described and justified
- Raw data should be presented in figures whenever informative to do so (typically when N per group is less than 10)
- For each experiment, you should identify the statistical tests used, exact values of N, definitions of center, methods of multiple test correction, and dispersion and precision measures (e.g., mean, median, SD, SEM, confidence intervals; and, for the major substantive results, a measure of effect size (e.g., Pearson's r, Cohen's d)
- Report exact p-values wherever possible alongside the summary statistics and 95% confidence intervals. These should be reported for all key questions and not only when the p-value is less than 0.05.

Please outline where this information can be found within the submission (e.g., sections or figure legends), or explain why this information doesn’t apply to your submission:

Statistical reporting method was described in the section Statistical analysis of Materials and Methods. The information about the statistical analysis of the confocal microscopy images is provided in the “Co-localisation analysis” section of Materials and Methods. The information about the statistical analysis of the RNAsseq data is provided in the “RNA-seq – analysis” section of Materials and Methods. The particular values for western blot analysis such as number of n used for the statistical analysis, precision measures, p-values are provided in the following figure legends: Figure 1D, Figure 3B, Figure 3F, Figure 3H, Figure 3M, Figure 3 –figure supplement 2B-D, Figure 4B-C, Figure 4F-G, Figure 4I, Figure 4 –figure supplement 1F-G, Figure 5B-C, Figure 5D, Figure 5F, 5G, Figure 5 – figure supplement 1G-H.

(For large datasets, or papers with a very large number of statistical tests, you may upload a single table file with tests, Ns, etc., with reference to sections in the manuscript.)

**Group allocation**

- Indicate how samples were allocated into experimental groups (in the case of clinical studies, please specify allocation to treatment method); if randomization was used, please also state if restricted randomization was applied
- Indicate if masking was used during group allocation, data collection and/or data analysis

Please outline where this information can be found within the submission (e.g., sections or figure legends), or explain why this information doesn’t apply to your submission:

The investigators were not blinded to allocation during experiments and outcome assessment. The statement with such information is provided in Materials and Methods chapter, section “Experimental Design”.

**Additional data files (“source data”)**

- We encourage you to upload relevant additional data files, such as numerical data that are represented as a graph in a figure, or as a summary table
- Where provided, these should be in the most useful format, and they can be uploaded as “Source data” files linked to a main figure or table
- Include model definition files including the full list of parameters used
- Include code used for data analysis (e.g., R, MatLab)
- Avoid stating that data files are “available upon request”

Please indicate the figures or tables for which source data files have been provided:

The source data file is provided for the RNAseq data analysis, presented in Figure 2, Figure 2 – figure supplement 1 and Figure 2 – figure supplement 2.

For data presented in Figure 4 and Figure 4 – figure supplement 1, and for data presented in Figure 5 and Figure 5 – figure supplement 1.
